# Chromosome-level genome assembly and annotation of the spottedtail morwong (*Cheilodactylus zonatus*)

**DOI:** 10.1038/s41597-025-06029-x

**Published:** 2025-11-04

**Authors:** Mengyang Chang, Kunpeng Shi, Wensheng Li, Wenhui Ma, Songlin Chen, Zhenxia Sha

**Affiliations:** 1 Institute of Aquatic Biotechnology, College of Life Sciences, Qingdao University, Qingdao, 266071 China; 2 Laboratory for Marine Fisheries Science and Food Production Processes, Qingdao National Laboratory for Marine Science and Technology, Qingdao, 266071 China; 3 Shandong Center of Technology Innovation for Biological Breeding of Premium Fish (Preparatory), Qingdao, 266071 China; 4 Laizhou Mingbo Aquatic Co., Ltd, Yantai, 261400 China; 5 State Key Laboratory of Mariculture Biobreeding and Sustainable Goods, Yellow Sea Fisheries Research Institute, Chinese Academy of Fishery Sciences, Qingdao, 266071 China

**Keywords:** Agricultural genetics, DNA sequencing

## Abstract

The spottedtail morwong (*Cheilodactylus zonatus*), belonging to the family Cheilodactylidae and the genus *Cheilodactylus*, is an important economic fish known for its delicate flesh and rich nutritional value. It mainly feeds on crustaceans and mollusks and is widely distributed in the western Pacific Ocean. However, the lack of effective reference genomes has seriously hindered further research on its biology and genomic breeding. Here, we present a high-quality chromosome-level genome assembly of *C. zonatus* using PacBio sequencing and Hi-C technologies. The assembled genome has a total length of 612.58 Mb with a contig N50 of 25.86 Mb. Approximately 97.8% of the assembly length was anchored to 24 pseudochromosomes. A total of 26,083 protein-coding genes were predicted, among which 23,393 genes (89.67%) were functionally annotated. The completeness of the assembly was estimated to be 98.2% by BUSCO assessment. As we know, this is the first reference genome in the family Cheilodactylidae, which will provide pivotal genomic resources for further biological and evolutionary studies of *C. zonatus*, and facilitate the development of genomic hybridization breeding in the artificial *C. zonatus* industry.

## Background & Summary

The family Cheilodactylidae, within the order Perciformes, comprises approximately five genera and around 26 species, with *Cheilodactylus zonatus* representing one of its most characteristic members^1^. However, no reference genome has yet been reported for any member of the family Cheilodactylidae. This species is widely distributed in the western Pacific, from Japan to the South China Sea. The *C. zonatus* has an oval, laterally compressed body, with the dorsal profile rising steeply behind the eyes and reaching its highest point near the origin of the dorsal fin before sloping downward^2^. It inhabits benthic zones around reef-sand interfaces, where it exhibits a stop-and-go swimming pattern. Individuals frequently rest above reef platforms, lie in wait to ambush prey, or use their elongated pectoral fin rays to probe sandy or muddy substrates in search of food, primarily benthic crustaceans^3^. Due to its distinctive taste and texture, *C. zonatus* possesses high economic value and holds promise as a potential candidate for aquaculture development.

In contrast, high-quality reference genomes have been successfully sequenced for several important fish species in recent years, including giant grouper (*Epinephelus lanceolatus*)^4^, leopard coral grouper (*Plectropomus leopardus*)^5^, and tomato hind (*Cephalopholis sonnerati*)^6^. These studies have provided a solid foundation for the conservation of aquaculture genetic resources, environmental adaptability research, and molecular breeding. With the widespread application of advanced genomic technologies, such as Single Molecule Real-Time sequencing (SMRT) and high-throughput chromatin conformation capture (Hi-C), chromosome-level genome assembly for non-model fish species has now become achievable.

In this study, we employed an integrated strategy of HiFi long reads, Hi-C, Iso-seq and RNA-seq sequencing technologies to assemble a high-quality genome of *C. zonatus*. This genome was 612.58 Mb with contig N50 of 25.86 Mb. Approximately 97.8% (598.6 Mb) of assembled sequences were placed into 24 pseudochromosomes with the support of Hi-C contact map. 26,083 protein-coding genes were predicted and 89.67% were functionally annotated. The Benchmarking Universal Single-Copy Orthologs (BUSCO)^7^ assessment of the assembly showed 3,336 (98.2%) BUSCOs was complete. This high-quality *C. zonatus* reference genome will provide an important genomic resource for genetic breeding and molecular mechanism related studies.

## Methods

### Sample preparation

The sample was collected in September 2024 from Laizhou Mingbo Aquatic Co., Ltd. and derives from a wild population in the South China Sea. The individual was dissected on ice, and then seven tissues (muscle, gills, liver, intestine, kidney, brain, and blood) were excised. After that, these tissues were immediately frozen in liquid nitrogen for nucleic acid extraction. During tissue extraction using scalpels, forceps, and modified scissors, the tissues and sampling tools were washed separately with phosphate-buffered saline (PBS, 1X) and ethanol in order to prevent contamination from external impurities or cross-contamination between different tissue types. The samples were stored at −80 °C. High molecular weight genomic DNA was extracted for Illumina and PacBio Hifi sequencing by TIANamp Genomic DNA Kit (Qiagen, Germany). Total RNA was isolated from all seven tissues using TRIzol reagent (Vazyme, China) for transcriptome sequencing.

### Illumina sequencing

The high-quality genomic DNA of muscle tissue was randomly fragmented into segments by the Covaris ultrasonic crusher to prepare a paired-end library with the inserted fragments of 350 bp using the Illumina DNA PCR-Free Prep kit (Illumina, USA). The constructed library was sequenced on Illumina Novaseq X platform (Illumina, USA). To ensure the quality of analysis, the raw reads were processed by fastp (v0.24.1)^8^ to filter out adapters and low-quality reads, and 38.27 Gb of clean data was obtained (Table 1).

**Table 1:** Statistical analysis of sequencing reads from Illumina and PacBio.

Libraries	Insert size (bp)	Clean data (Gb)	Reads number	Read length (bp)	Sequence coverage (×)
Illumina reads	350	38.27	127,575,305	150	64.86
PacBio reads	15,000	55.76	3,465,867	17,031 (mean)	94.51

### PacBio HiFi sequencing

The high-quality genomic DNA of muscle tissue was randomly fragmented into 15–17 kb to build a PacBio HiFi library by using SMRTbell Express Template Prep Kit 2.0 (Pacific Biosciences, USA) according to the manufacturer’s instructions. The generated library was sequenced on the Pacbio Sequel II platform (Pacific Biosciences, USA) using SMRT (single molecule real-time) circular consensus sequencing (CCS) technology with SMRT Cell 8 M Tray. After removing adapters in polymerase reads, a total of 55.76 Gb of HiFi clean data was generated, with an average read length of 17,031 bp (Table 1).

### Hi-C sequencing

Muscle tissue from the foot of *C. zonatus* was used to construct the Hi-C library according to a standard protocol^9^ with several modifications. Briefly, the tissue was ground in liquid nitrogen and cross-linked with a 4% formaldehyde solution to preserve the 3D structure of the DNA. After overnight digestion with the 4-cutter restriction enzyme MboI, the DNA ends were labeled with biotin-14-dCTP. The subsequent steps involved blunt-end ligation of the cross-linked fragments, re-ligation of the proximal chromatin DNA, and reverse crosslinking of the nuclear complexes. DNA was then purified by using phenol-chloroform extraction. After removing biotin from non-ligated fragment ends and repairing of sheared fragment ends, biotin-labeled Hi-C samples were enriched, ligated with sequencing adapters, and amplified by polymerase chain reaction (PCR, 12–14 cycles). Hi-C library preparation and sequencing were performed with muscle tissue using a Dovetail Hi-C Core Kit (Dovetail Genomics, USA) following protocol instructions. The qualified library was sequenced on the Illumina Hiseq platform. After adapter removal and filtering of low-quality reads using Trim Galore (v0.6.10, https://github.com/FelixKrueger/TrimGalore), 70.8 Gb of clean data were obtained, with a Q20 score of 98.52% (Table 2).

**Table 2:** Statistical analysis of sequencing data from Hi-C.

Type	Data
Raw paired reads	239,294,110
Raw Base (bp)	71,788,233,000
Clean Base (bp)	70,804,734,207
Effective Rate (%)	98.63%
Q20 (%)	98.52
Q30 (%)	94.88
GC Content (%)	45.16

### RNA-seq sequencing

For transcriptome sequencing, muscle, gills, liver, intestine, heart, skin, and and whole blood of *C. zonatus* were collected to construct sequencing libraries. Next-generation RNA-seq libraries were constructed with the NEBNext® UltraTM RNA Library Prep Kit for Illumina (NEB, USA) according to the manufacturer’s suggestions and sequenced on the Illumina Novaseq X platform (Illumina, USA) for PE150. The third-generation full-length RNA sequencing library was constructed by the Kinnex PCR cDNA Synthesis Kit (Pacific Biosciences, USA) and sequenced on PacBio Sequel II platform (Pacific Biosciences, USA). A total of 17.71 Gb of Isoform-Sequencing (Iso-Seq) reads was obtained (Table 3).

**Table 3:** Statistical analysis of RNA-seq from different tissues.

Sample	Platform	Raw Reads	Clean Reads	Q20 (%)	Q30 (%)	GC (%)
Muscle	Illumina	44,335,212	42,803,966	96.95	91.58	35.06
Gills	Illumina	41,498,594	40,662,494	97.34	92.2	34.3
Liver	Illumina	45,804,886	44,959,106	97.72	93.25	36.73
Intestine	Illumina	44,078,548	42,905,486	97.28	92.44	36.17
Kidney	Illumina	45,450,476	43,895,912	97.38	92.66	35.6
Brain	Illumina	42,146,312	40,323,936	97.4	92.75	37.09
Mix	PacBio	10,171,245				

### Genome survey

**Fig. 1:**
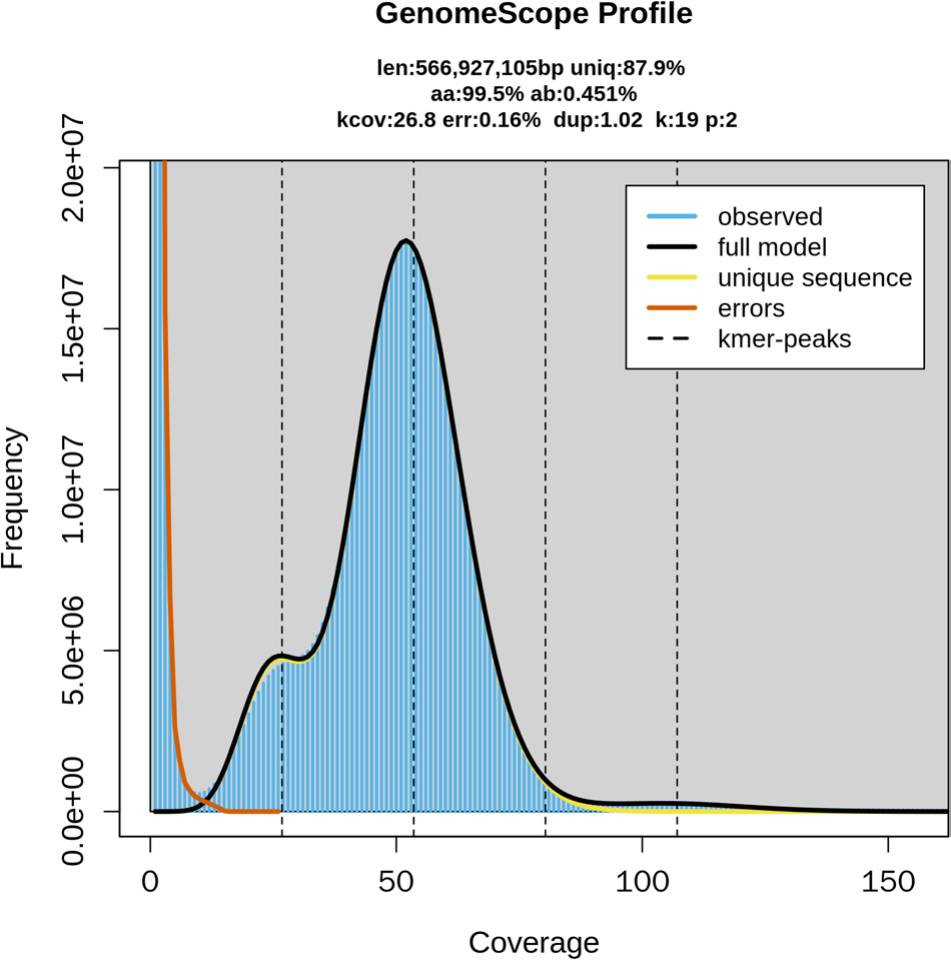
Genome survey at 21-mer of *C. zonatus*.

Genomic surveys were conducted to assess the genomic characteristics, which includes genome size, heterozygosity, and replication rates, so as to determine the amount of subsequent sequencing data. The software Jellyfish (v 2.2.10)^10^ was utilized with parameters set as ‘-m 19 -s 20 G -C’ to count k-19mers using the Illumina-generated short reads as input. The generated histogram was then employed as an input file for GenomeScope (v2.0)^11^ to estimate the genome characteristics. It was found that the genome size and heterozygosity of *C. zonatus* were approximately 566.92 Mb and 0.45%, respectively (Fig. 1).

### Genome assembly

**Fig. 2:**
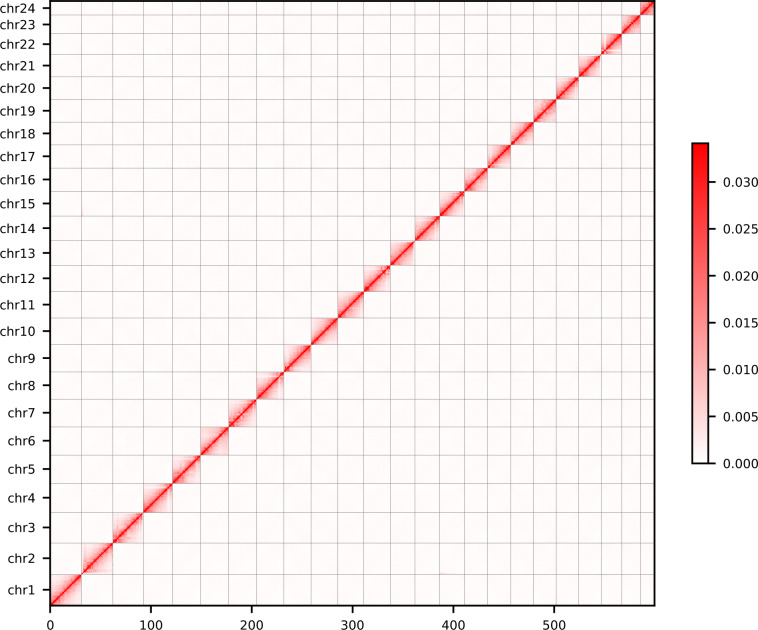
Genome-wide heatmap of Hi-C interactions among 24 chromosomes in *C. zonatus*. Various colors represent different interaction frequencies of Hi-C links, and the aggregated color blocks represent the interaction frequencies between individual chromosomes.

**Fig. 3:**
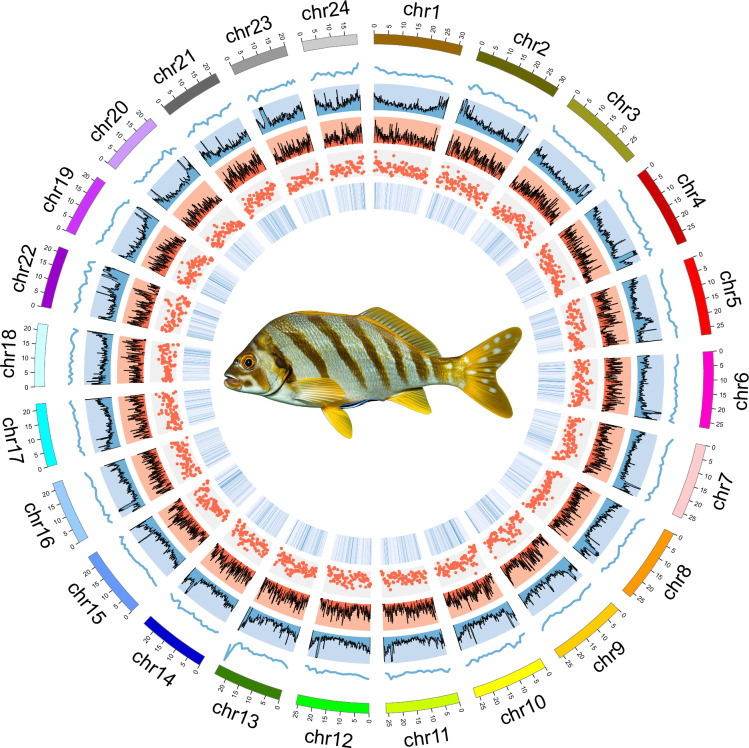
Circos plot of genomic features in *C. zonatus*. Each track from outer to inner represents the chromosome length (Mb), and the distribution of GC content, DNA element, short interspersed nuclear element (SINE) density, long interspersed nuclear element (LINE) density, and protein-coding genes with a sliding window size of 1 Mb.

Based on PacBio HiFi and Hi-C reads, the genome was *de novo* assembled by Hifiasm (v0.25.0)^12^ in ‘Hi-C Intergrated Assembly’ mode with default parameters. Then, purge_dups (v1.2.6)^13^ was used to remove haplotypic and heterozygous duplication from the de novo assembly, resulting in a total length of 612.58 Mb. To generate the chromosome-level genome of *C. zonatus*, the 121.66 × Hi-C data obtained from sequencing, the contigs sequences obtained from the assembly were anchored to the chromosome level using Haphic (v1.0.7)^14^ (Fig. 2). The final *C. zonatus* genome size was 612.58 Mb, and a total of 598.6 Mb (97.8%) sequences were anchored to 24 chromosomes, of which the longest and shortest were 31.22 Mb and 13.63 Mb, respectively (Table 4, Fig. 3).

**Table 4:** Assembly statistics of *C. zonatus* genome.

Statistic	Contig (bp)	Scaffold (bp)
Total Number	111	86
Total Length	612,588,814	612,588,014
Average Length	5,518,810	5,947,464
Max Length	31,225,458	31,225,458
N50 Length	25,860,851	25,860,851
N50 Number	11	11
N90 Length	20,406,750	20,406,750
N90 Number	22	22

### Annotation of repetitive elements

A comprehensive repeat library for the *C. zonatus* genome was constructed by combining Extensive *de novo* TE Annotator (EDTA, v2.2.2)^15^ with RepeatModeler (v2.0.6)^16^. Based on this *de novo* repeat library, repetitive elements were predicted and classified by RepeatMasker (v4.1.8)^17^. The repeat annotation results showed that repetitive elements accounted for 33.72% of the assembled genome. Among these, DNA transposons were the most abundant (13.13%), followed by long interspersed nuclear elements (LINEs, 6.17%) and unclassified repeats (10.71%). Other types included long terminal repeats (LTRs, 1.63%), Penelope elements (0.17%), short interspersed nuclear elements (SINEs, 0.61%), and DIRS elements (0.62%). Additionally, tandem repeats such as satellites (0.43%), simple repeats (0.14%), and low-complexity regions (0.11%) were also identified (Table 5, Fig. 3).

**Table 5:** Classification of repetitive sequences in *C. zonatus* genome.

Type			Count	Length (bp)	% of Genome
Dispersed repeats	DNA transposons		760860	78619798	13.13
	Retroelements	DIRS	49652	3696398	0.62
		LTR	110997	9738616	1.63
		LINE	339366	36929206	6.17
		Penelope	12388	1042904	0.17
		SINE	34362	3627680	0.61
	Unclassified		619635	64114833	10.71
Tandem repeats	Simple repeats		11519	816219	0.14
	Low complexity		11407	601427	0.11
	Satellite		17133	2590956	0.43
Total			1967319	201778037	33.72

### Noncoding RNA (ncRNA) annotation

Ribosomal RNAs (rRNAs) were predicted by using Barrnap (v0.9, https://github.com/tseemann/barrnap) with default parameters. For the prediction of transfer RNAs (tRNAs), tRNAscan-SE^18^ was utilized. Small nuclear RNAs (snRNAs) and microRNAs (miRNAs) were predicted by Infernal (v1.1.4)^19^ based on genome alignment with the Rfam database (v14.8)^20^. In total, 2,041 non-coding RNAs (ncRNAs) were annotated, which included 342 rRNAs, 1,013 tRNAs, 128 snRNAs, 9 long non-coding RNAs (lncRNAs), and 549 miRNAs (Table 6).

**Table 6:** Classification of ncRNAs in *C. zonatus* genome.

Type		Copy Number	Average Length (bp)	Total Length (bp)	% of Genome
tRNA		1013	73.08	74030	0.12371219
miRNA		549	80.71	44308	0.07395285
lncRNA		9	1200	10800	0.00001805
snRNA	CD-box	29	120	3480	0.00000581
	HACA-box	17	230	3910	0.00000653
	scaRNA	1	150	150	0.00000025
	splicing	81	160	12960	0.00002165
rRNA	5S	327	115	37605	0.06282348
	5.8S	5	160	800	0.00000134
	18S	5	1700	8500	0.0000142
	28S	5	4250	21250	0.00003547

### Prediction and functional annotation of protein-coding genes

**Fig. 4:**
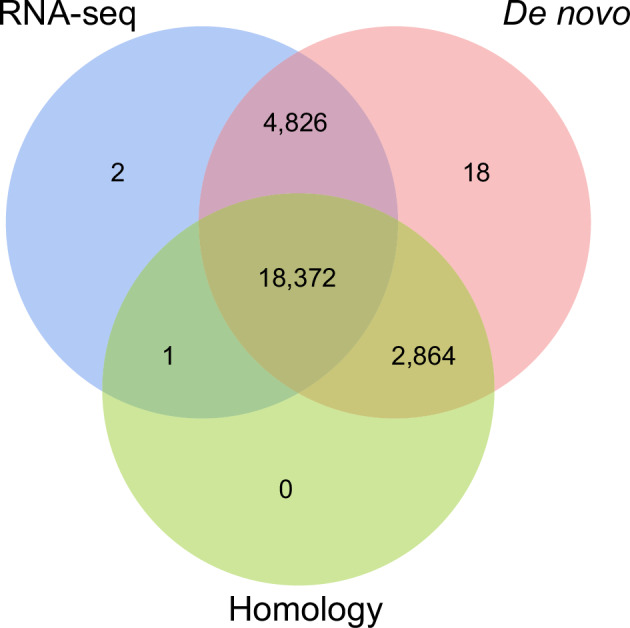
Venn diagram of *C. zonatus* gene structure prediction through three strategies.

**Fig. 5:**
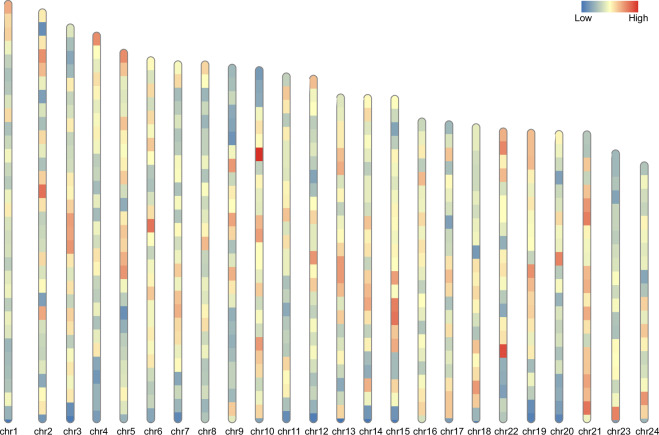
Gene density of *C. zonatus* each chromosome within 1 Mb sliding windows.

**Fig. 6:**
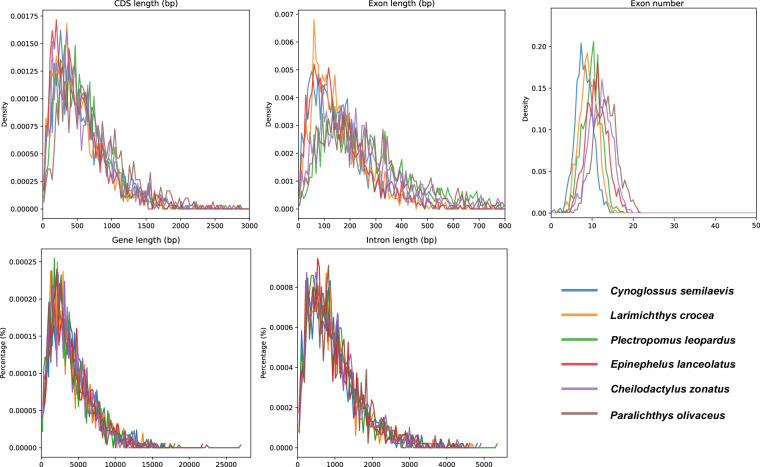
The Gene length, CDS length, intron length, exon length, exon number per gene in *C. zonatus* compared with five other species.

Protein-coding genes structure prediction was performed by integrating de novo prediction, homology-based prediction, transcriptome-based prediction, and prediction supported by other evidence in repeat-masked genome (Fig. 4). *De novo* prediction was used to predict gene structure (e.g. codon frequency, exon-intron distribution) using software Augustus (v3.3.3)^21^, GlimmerHMM (v3.04)^22^, GeneMark (v4.71_lic)^23^, SNAP (v2006-07-28)^24^ and BRAKER2 (v2.1.8)^25^. For homology prediction, reference protein-coding gene sets of six closely related species, including *Cynoglossus semilaevis* (GCA_000523025.1), *Epinephelus lanceolatus* (GCA_005281545.1), *Larimichthys crocea* (GCA_000972845.2), *Paralichthys olivaceus* (GCA_024713975.2), *Plectropomus leopardus* (GCA_008729295.2), and *Cephalopholis sonnerati* (GCA_043388385.1), were downloaded from NCBI databases to generate a homology-based protein-coding gene set. The homology-based coding protein sequence sets were aligned with the genomes and predicted with gene structure information using TblastN (v2.7.1)^26^, GeneWise (v2.4.1)^27^, and GenomeThreader (v1.7.3)^28^. Other predictions supported by evidence are the prediction of gene structure by Blat alignment using EST or cDNA data from homologous species. In transcriptome-based prediction, RNA-seq data from different tissues were compared to the genome using HISAT2 (v2.2.1)^29^ and Samtools (v1.19)^30^, identifying exonic regions and splice sites using default parameters. Transcript assembly was performed using StringTie (v2.2.1)^31^ against the results. Combining the above prediction results, the gene sets obtained from the various methods of prediction were integrated into a non-redundant and more complete gene set using the EVidenceModeler (EVM, v2.1.0)^32^ integration software. Lastly, the final non-redundant reference gene set was obtained by using PASA (v2.5.3)^33^, combining the transcriptome assembly results, correcting the annotation results of EVM, and adding information such as UTR and variable shear to obtain the final non-redundant reference gene set. Finally, 26,083 protein-coding genes in the genome of *C. zonatus* were predicted, with an average gene length of 15,094.75 bp. The average lengths of CDSs, exons, and introns of each gene were 1,506.73, 1,989.42 bp, and 252.77 bp, respectively (Table 7). To visualize gene distribution, we plotted the density of genes on 24 chromosomes with a window of 1 Mb in length using RIdeogram (v0.2.2) in R (Fig. 5). Meanwhile, the genomic features of *C. zonatus*, such as CDS, gene, exon and intron length distributions, were similar to those of homologous species (Fig. 6).

**Table 7:** Statistical results of the gene structure annotation in *C. zonatus* genome.

Method	Software	Species	Gene Number	Gene length (bp)	CDS length (bp)	Average intron length (bp)	Average exon length (bp)	Exon per gene
*De novo*	Augustus		29,184	10,087.35	1,489.70	223.45	1,498.87	6.74
	BRAKER2		32,165	10,181.22	1,478.66	215.84	2,265.39	6.98
	GlimmerHMM		84,963	7,905.64	741.22	206.11	2,933.04	3.62
	GeneMark		64,508	4,358.92	829.46	200.25	1,099.33	4.28
	SNAP		77,842	3,057.49	538.19	231.74	1,918.21	3.35
RNA-seq	HISAT2 & StringTie		20,103	20,738.41	2,226.98	454.91	2,626.65	9.61
	PASA		29,996	21,091.46	1,217.94	413.28	2,884.11	7.33
Homology	MetaEuk	*C. semilaevis*	24,161	6,705.35	1,046.70	274.45	1,614.45	7.4
		*E. lanceolatus*	23,639	7,730.76	1,174.61	232.39	1,576.56	11.15
		*L. crocea*	24,309	6,519.86	981.48	224.53	1,600.26	9.45
		*P. olivaceus*	22,207	6,971.27	1,086.48	239.7	1,628.24	12.6
		*P. leopardus*	23,892	3,771.01	809.98	289.93	1,619.86	11.82
Final		—	26,083	15,094.75	1,506.73	252.77	1,989.42	7.58

**Fig. 7:**
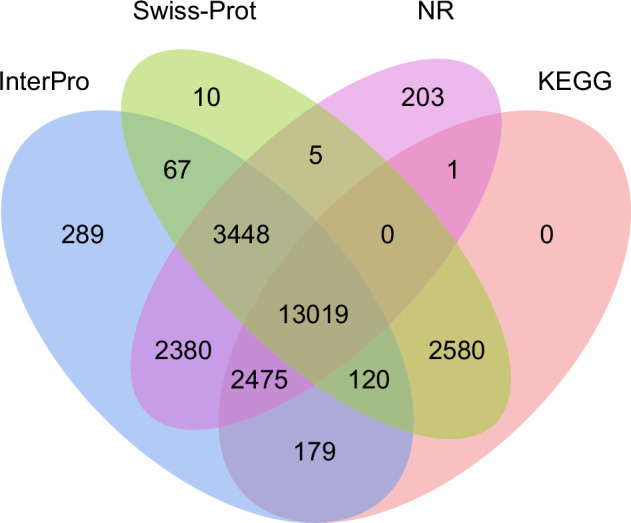
Venn diagram of *C. zonatus* functional gene annotation based on four of the eight databases.

Functional annotation of the protein-coding genes was carried out using the Funannotate pipeline (v1.8.16), based on multiple databases, including Clusters of Orthologous Groups of Proteins (COG)^34^, eggNOG^35^, Gene Ontology (GO)^36^, InterPro^37^, and Pfam^38^. Subsequently, functional annotation was performed by aligning the protein-coding genes to the National Center for Biotechnology Information (NCBI) non-redundant protein (Nr) and Swiss-Prot databases^39^ using Diamond (v2.1.8.162)^40^, with an E-value cutoff of 1e⁻⁵. Additionally, Kyoto Encyclopedia of Genes and Genomes (KEGG) annotations were obtained by eggNOG-mapper (v2.1.12)^41^. A total of 23,393 genes (89.6%) of the predicted proteins were functionally annotated, of which 17,441 proteins were annotated in the Swiss-Prot database, 22,758 proteins in the NR database, 19,221 proteins in the InterPro database, and 16,221 proteins in the Pfam database (Fig. 7; Table 8).

**Table 8:** Summary of the functional gene annotation in *C. zonatus* genome.

Database	Number	Percent(%)
Total	26,083	100
COG	18,035	69.21
eggNOG	18,982	72.86
GO	16,004	61.44
InterPro	19,211	73.78
KEGG	14,157	54.32
Pfam	16,221	62.18
Swiss-Prot	17,441	66.94
Nr	22,758	87.27
Annotated	23,393	89.88
Unannotated	2,690	10.12

## Data Records

The genomic Illumina, PacBio, Hi-C, and transcriptomic sequencing data were deposited in the Sequence Read Archive (SRA) at NCBI under the accession numbers SRP583805^42^.

The final chromosome assembly was deposited in the GenBank under the accession GCA_050575295.2^43^.

The annotation files have been uploaded to the figshare database, with the DOI number: 10.6084/m9.figshare.28953329^44^.

## Technical Validation

### Quality evaluation of the extracted nucleic acid

The concentrations of DNA and RNA were measured by Nanodrop 2000 spectrophotometers (Thermo Fisher Scientific, USA) and 5400 Fragment Analyzer system (Agilent Technologies, USA). Agarose gel electrophoresis was applied to evaluate the integrity and purity of nucleic acid.

### Evaluation of the genome assembly and annotation

The present study utilized a total of 94.51× PacBio HiFi reads and 121.66× Hi-C reads to ensure a high-quality genome assembly. Prior to this, we conducted a 19-mer distribution analysis to estimate the genome size based on 64.86× Illumina reads. To assess the quality of *C. zonatus* genome assembly, we adopted three methods as follows. Firstly, we used Merqury (v1.3)^45^ to estimate the base-level accuracy and completeness based on k-mer counts generated from Illumina reads, resulting in a QV of 54.73 (Table 9). Secondly, Clipping Reveals Assembly Quality (CRAQ, v1.09)^46^ was used to assess the accuracy of genome assembly based on PacBio HiFi reads and Illumina reads, resulting in a R-AQI (assembly quality indicator) of 94.72% and a S-AQI of 99.28% (Table 9). Thirdly, we mapped Illumina clean reads to *C. zonatus* genome using bwa (v0.7.17-r1188)^30^. The statistical result from samtools (v1.9)^47^ showed the genome mapping rate and the coverage rate were 99.24% and 99.97%, respectively (Table 9).

**Table 9:** Assessment metrics of *C. zonatus* genome assembly and annotation.

	Type		Result
Genome accuracy and completeness	Mapping short-reads rate		99.24%
	Genome coverage rate with short-reads		99.97%
	Quality value scores (QVs)		54.73
	BUSCOs	Complete BUSCOs (C)	98.2% (3,574)
		Complete and single-copy BUSCOs (S)	97.5% (3,549)
		Complete and duplicated BUSCOs (D)	1.4% (51)
		Fragmented BUSCOs (F)	0.4% (15)
		Missing BUSCOs (M)	0.7% (25)
	CRAQ	R-AQI	94.72%
		S-AQI	99.28%
Annotation quality	Complete BUSCOs (C)		95.9% (3,491)
	Complete and single-copy BUSCOs (S)		95.2% (3,465)
	Complete and duplicated BUSCOs (D)		1.2% (44)
	Fragmented BUSCOs (F)		0.8% (29)
	Missing BUSCOs (M)		3.4% (124)

The completeness of the *C. zonatus* genome assembly was evaluated using BUSCO (v5.2.2)^48^ with the *actinopterygii_odb10* database comprising 3,640 single-copy orthologs. The assessment revealed that 98.2% (3,574) of BUSCOs were complete, including 97.5% (3,549) single-copy and 1.4% (51) duplicated BUSCOs. Additionally, 0.4% (15) were fragmented and only 0.7% (25) were missing, indicating that the assembled genome has high completeness and integrity. These results collectively revealed the high quality of *C. zonatus* genome assembly.The accuracy of gene annotation was evaluated using BUSCO (v5.2.2) on the basis of actinopterygii_odb10 database containing 3,640 BUSCOs. The results showed that 3,491 (95.9%) complete BUSCOs, containing 3,465 (95.2%) single-copy and 44 (1.2%) duplicated BUSCOs, were detected, 29 (0.8%) fragmented BUSCOs and 124 (3.4%) missing BUSCOs were identified.

## Data Availability

The detailed parameters were not specified in this study. All bioinformatics software and tools were employed strictly following their respective user manuals and standard protocols. No custom codes or scripts were developed or applied during the course of this study.
